# Non-human Primate *Macaca mulatta* as an Animal Model for Testing Efficacy of Amixicile as a Targeted Anti-periodontitis Therapy

**DOI:** 10.3389/froh.2021.752929

**Published:** 2021-11-05

**Authors:** Qin Gui, Denver J. Lyons, Janina Golob Deeb, B. Ross Belvin, Paul S. Hoffman, Janina P. Lewis

**Affiliations:** ^1^Philips Institute for Oral Health Research, Virginia Commonwealth University, Richmond, VA, United States; ^2^Department of Periodontics, Virginia Commonwealth University, Richmond, VA, United States; ^3^Department of Medicine, Division of Infectious Diseases and International Health, University of Virginia, Charlottesville, VA, United States; ^4^Department of Microbiology and Immunology, Virginia Commonwealth University, Richmond, VA, United States; ^5^Department of Biochemistry, Virginia Commonwealth University, Richmond, VA, United States

**Keywords:** amixicile, anaerobic bacteria, microbiome, periodontitis, non-human primates (NHP), targeted antibiotic

## Abstract

Periodontitis is an inflammatory condition triggered by selected oral microbiota; thus treatment strategies should be aimed at reducing the abundance of the pathogenic bacteria. An obstacle to preclinical testing of such strategies is the availability of reliable animal models. Here, a non-human primate (NHP), *Macaca mulatta*, was used to examine the effectiveness of a novel antimicrobial, amixicile, which inhibits pyruvate–ferredoxin oxidoreductase (PFOR) present in anaerobic bacteria. Animals were assessed for their periodontal health, including radiography, clinical attachment loss (CAL), presence of plaque (PI), bleeding on probing (BOP) and pocket depth (PD), and sampled for saliva, gingival crevicular fluid (GCF), and subgingival plaque to determine their baseline clinical status. Amixicile was then administered for 2 weeks (40 mg/kg/day) and the animals were monitored for periodontal health immediately after the antibiotic treatment, then at 1 month-, 3 months-, and 6-months posttreatment. Microbial species present in plaque and saliva were determined through 16S rDNA sequencing. Baseline assessment of the microbiome has shown a significant proportion of bacteria belonging to the *Streptococcus, Haemophilus, Porphyromonas, Gemella*, and *Fusobacterium* genera. The abundance of *Porphyromonas* and *Fusobacterium* was reduced following treatment with amixicile, whereas that of *Escherichia, Haemophilus*, and *Gemella* were elevated. CAL, PD, and BOP were also significantly reduced following the treatment. In conclusion, the NHP model proves useful for preclinical studies of strategies targeting selected members of the oral microbiome. We show that amixicile reduces the levels of anaerobic bacteria under *in vivo* conditions, correlating with a reduction in CAL, PD, and BOP, thus validating its usefulness as an antimicrobial strategy.

## Introduction

Although rodent models provide useful information for many of the oral bacteria–host interactions, they are inadequate to determine the efficacy of an antimicrobial agent to treat periodontitis in humans [1, 2]. We examined the efficacy of amixicile, a newly developed inhibitor of pyruvate ferredoxin oxidoreductase (PFOR), which is an enzyme critical for energy generation in anaerobic bacteria. In contrast, aerotolerant bacteria and eukaryotic cells use Pyruvate Dehydrogenase (PDH) that is not inhibited by amixicile. Previous studies of amixicile have shown promising outcomes in specific inhibition in the growth of anaerobic bacteria in both *in vitro* and *ex vivo* microbiomes [3, 4]. Suitability of amixicile was further supported by data showing its minimal effect on gut microbiota and its ability to concentrate at sites with inflammation [5, 6]. All these characteristics suggest that amixicile may be an antimicrobial for the treatment of periodontitis. So far, amixicile has been tested in our laboratory in settings that did not allow for the assessment of its efficacy on the microbiome when present in its natural niche, the subgingival pocket [3, 4, 7]. A major challenge for the assessment of targeted microbial therapies on the oral microbiome is the lack of a suitable animal model. Previous studies have shown that non-human primate (NHP), *Macaca mulatta* model provides significant similarity to the oral microbiome composition of the human [1, 8]. In addition, anatomical similarities, the clinical presentation of the disease, periodontal structures, and host immune response make the NHP model optimal for the evaluation of therapeutics in the development for the treatment of human periodontitis. Based on the above, we tested the suitability of the NHP model with naturally occurring periodontitis to examine the efficacy of amixicile as a potential treatment for periodontal disease.

While most of the antibiotics used in dentistry are broad spectrum and inhibit the growth of most bacteria through binding to the ribosome and inhibiting protein synthesis (azithromycin, clindamycin, and tetracycline) or inhibiting cell wall synthesis (amoxicillin), only metronidazole specifically inhibits anaerobic bacteria [9]. However, metronidazole is associated with several adverse effects including appetite loss, yeast infection, dizziness, headache, nausea, metallic taste, neuropathy, pancreatitis, encephalopathy, and has low solubility that limits its utility [10]. Thus, an antimicrobial agent with specific inhibition of anaerobic bacteria with minimal undesirable side effects remains elusive. Here, we performed a pilot study using an NHP animal model to test the potential of amixicile for the reduction of perio-pathogenic bacteria in the treatment of periodontitis.

## Materials and Methods

### Animals

Two male non-human rhesus primates, *Macaca mulatta* were used in the study. Animal G was 15 years old and weighed 7.95 kg while Animal T was 16 years old and weighed 12.09 kg at the initial exam. Both animals were systemically healthy and their dentitions were free of caries or endodontic pathology. They were housed at the animal facility of VCU in extra-large enclosures, provided with social and environmental enrichment, and fed a diet of kibble (Monkey Chow, Purina). For the duration of the study, the diet of the animals was changed to a soft one, and means of mechanical oral hygiene were reduced to allow for plaque accumulation. The animals were treated twice daily for 14 days with 20 mg/kg/animal of amixicile encased in a marshmallow.

### Clinical Examination and Sample Collection

The animals were placed under general anesthesia by injection of ketamine (10 mg/kg) followed by intubation and administration of 2% isoflurane at 2 L/min and 100% oxygen at 1 L/min.

**Figure 1:**
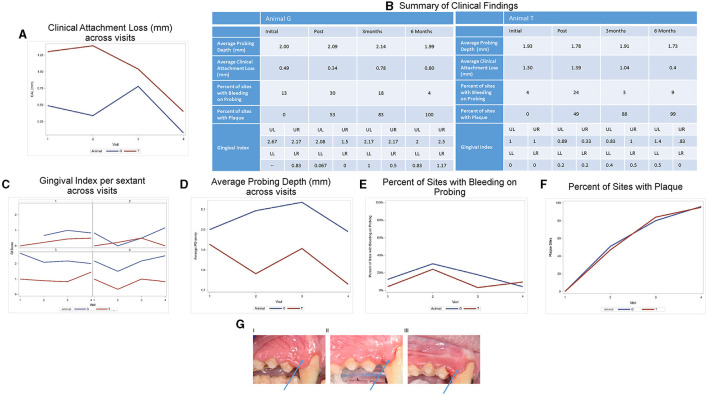
Clinical characteristics of animals. **(A)** Clinical attachment loss (CAL) at various times pre- and posttreatment. **(B)** Summary of clinical characteristics. Detailed analysis of clinical characteristics assessed: **(C)** gingival index (GI), **(D)** average probing depth [PD (shown in mm)], **(E)** average bleeding on probing [BOPpresent/absent], **(F)** number of sites with plaque. Two animals and 12 sites were examined. **(G)** Effect of amixicile on GI at various times post-treatment. Clinical photos of Animal G pre-treatment (I), 3 months posttreatment (II), and 6 months posttreatment (III). Arrow points to a significant reduction of edema in the interproximal papillae.

**Figure 2:**
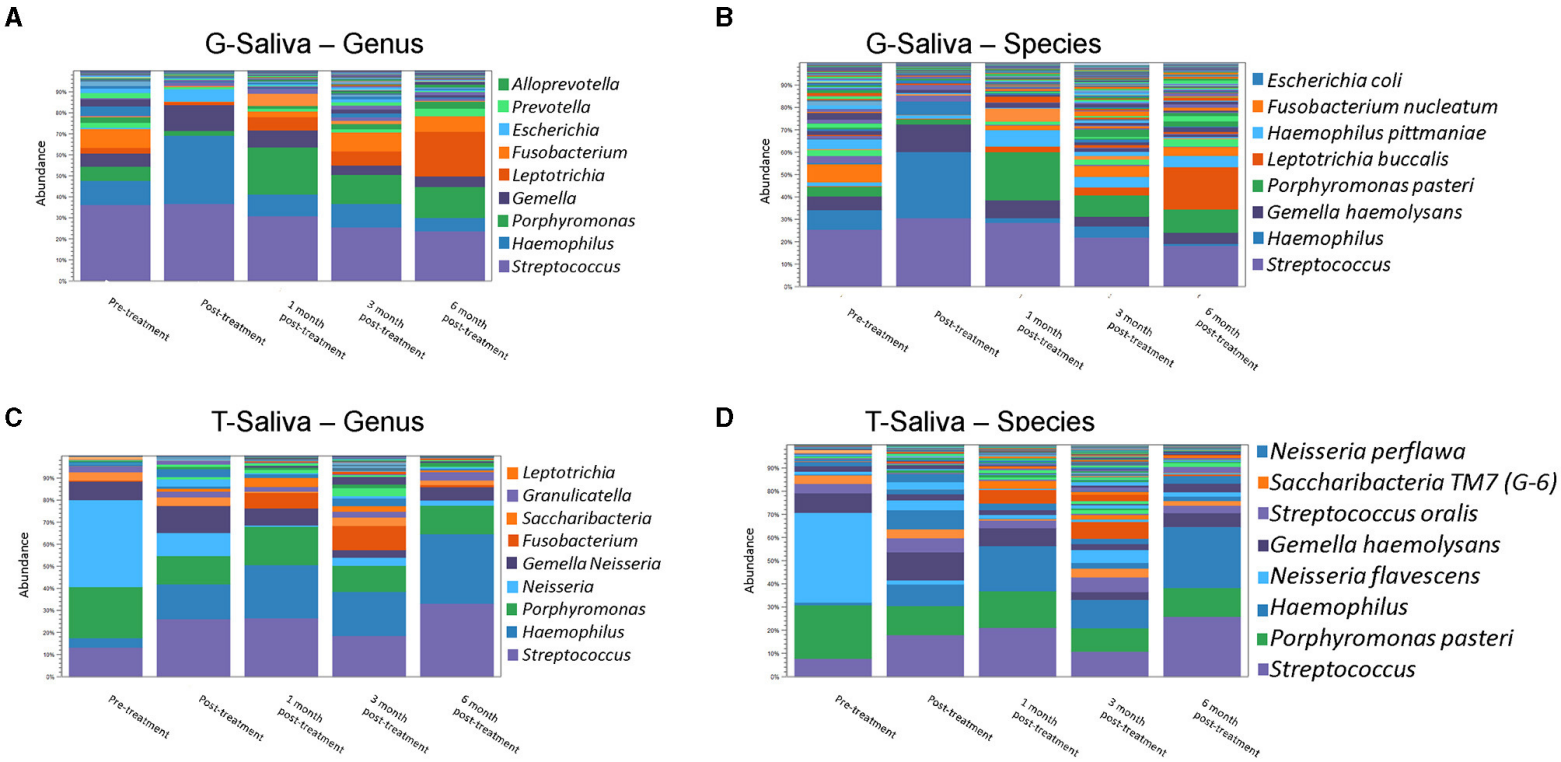
Amixicile reduces the abundance of anaerobic bacteria in the salivary microbiome. Data from analysis done at the level of genus **(A,C)** and species **(B,D)**. Two animals G **(A,B)** and T **(C,D)** were examined.

Clinical photographs and a comprehensive periodontal examination were performed during four visits: (1) baseline (pretreatment, visit 1), (2) posttreatment (visit 2), (3) 3-months posttreatment (visit 3), (4) 6-months posttreatment (visit 4) (Supplemental Figure 1). Periapical radiographs were taken at visit 1. Periodontal exam included recordings of pocket depth (PD), free gingival margin (FGM), bleeding on probing (BOP), and the presence of plaque at four sites (mesiobuccal (MB), buccal (B), distobuccal (DB), and lingual (L) on each tooth. Clinical attachment loss (CAL) was calculated from PDs and FGM values. Diagnosis of periodontitis was determined using CAL and supported by radiographic bone loss on both animals (Figure 1A). Photographs were used to assign a modified gingival index (GI) [11] score to each sextant for each exam. The scoring was as follows: GI 0 = pale pink to pink, knife-edge margin, positive architecture; GI 1 = slightly reddish, slight marginal edema, clear exudate, no bleeding on probing (BOP); GI 2 = red to bluish-red, glazy, marginal edema, BOP; GI 3 = markedly red to bluish, edematous, BOP/spontaneous bleeding. Photographs were randomized and each sextant was scored with a single value by three independent examiners and averaged to assess the GI of each sextant for the four exams (Figure 2).

Saliva samples were collected using five cotton swabs equally representing all areas of the mouth by swabbing the entire oral cavity. The collected cotton swabs were placed in microcentrifuge tubes containing 500 μl of phosphate-buffered saline (PBS, pH 7.2) and RNAlater solution (Invitrogen, Waltham, Massachusetts, USA).

Gingival crevicular fluid (GCF) and plaque samples for microbiological analysis were harvested from 12 selected sites in all four exams exam to correlate the findings. For subgingival plaque collection, a Nevi 2 periodontal scaler (SCNEVI29E2, Hu-Friedy, Chicago, Illinois, USA) was inserted to the base of the attachment, and the plaque was collected from the subgingival area and stored in RNA later at −80°C. For GCF collection, a Periostrip paper (Periopaper Gingival Crevicular Fluid Collection Strip, Fisher Scientific International, Pittsburgh, Pennsylvania, USA) was inserted into the periodontal sulci of interest and left for 30 s or until visibly saturated. The samples were collected into PBS and stored at −80°C.

### Microbiological Analysis

Plaque samples were processed individually whereas aliquots of saliva samples were pooled together prior to analysis. Genomic DNA (gDNA) was isolated using 200 μl of the collected samples with the PureLink™ Microbiome DNA purification kit (Thermo-Fisher Scientific) according to instructions of the manufacturer.

#### Metagenomic Library Generation and 16S rDNA Sequencing

Bacterial 16S ribosomal DNA (rDNA) amplification and library construction were done using the Zymo Research Quick-16S^TM^ NGS Library Prep Kit (Zymo Research, Irvine, California, USA) according to the low DNA input protocol. Briefly, reactions were set up in a 96-well “Targeted Plate” and the V3–V4 region of rRNA genes were amplified with the V3–V4 primers and the Quick-16STM qPCR premix. Twenty five cycles (and more, if required) at the profile 95°C for 10 min, 95°C for 30 s, 55°C for 30 s, and 72°C for 3 min were used for amplification. Following cooling at 4°C, the samples were transferred to the collection plate and PCR primers/dNTPs were degraded enzymatically. Finally, the samples were transferred to a “barcoded plate” where index primers for multiplexing of the samples were added. The barcodes were added using five PCR cycles consisting of 95°C for 10 min, 95°C for 30 s, 55°C for 30 s, and 72°C for 3 min. The library was then pooled in equimolar amounts and purified using the MagBead kit components (Zymo Research, Irvine, California, USA). The final 16S rDNA library was sequenced with the MiSeq Reagent Kit v3 (600-cycle) with pair end-setting and 2 × 250 bp on the Illumina MiSeq platform (Illumina, San Diego, California, USA). Sequencing was performed at the VCU Genomics and Microbiome Core, Richmond, Virginia, USA. Following sequencing, the samples were deconvoluted, barcodes were trimmed, and short sequences (<100bp) were removed.

#### Metagenomic Data Processing

The raw read sequences were analyzed with CLC Workbench (version 12; Qiagen, Venlo, Netherlands) equipped with the Microbial Genomics Module plugin (version 2.0; Qiagen). The paired end reads were merged into one high-quality representative by settings of CLC Workbench (mismatch cost = 1, minimum score = 25, gap cost = 4, maximum unaligned end mismatches = 5). The parameter settings for the quality trimming were as follows: trim using quality scores, limit = 0.05; trim ambiguous nucleotides, the maximum number of ambiguities = 2. Operational taxonomic unit (OTU) clustering and taxonomic assignment were carried out with the reference sequences from the Human Oral Microbiome Database (HOMD, 16S rRNA gene reference sequence [16S rRNA refSeq] Version 15.2) at a level of similarity of 97% of OTU. To avoid analysis of spurious OTUs, only those with more than 10 reads in at least one sample were kept.

### Statistical Analysis

Due to the limited sample size and nature of this pilot study, statistical data analysis will be primarily descriptive in nature with the intent of providing information to power future studies rather than to determine statistical significance. The average values were calculated for PDs, CAL, BOP, and the presence of plaque for each animal and each exam. Clinical indices for selected sites were compared with microbiological findings on an individual basis.

## Results

### Amixicile Reduces Clinical Characteristics of Periodontitis

**Figure 3:**
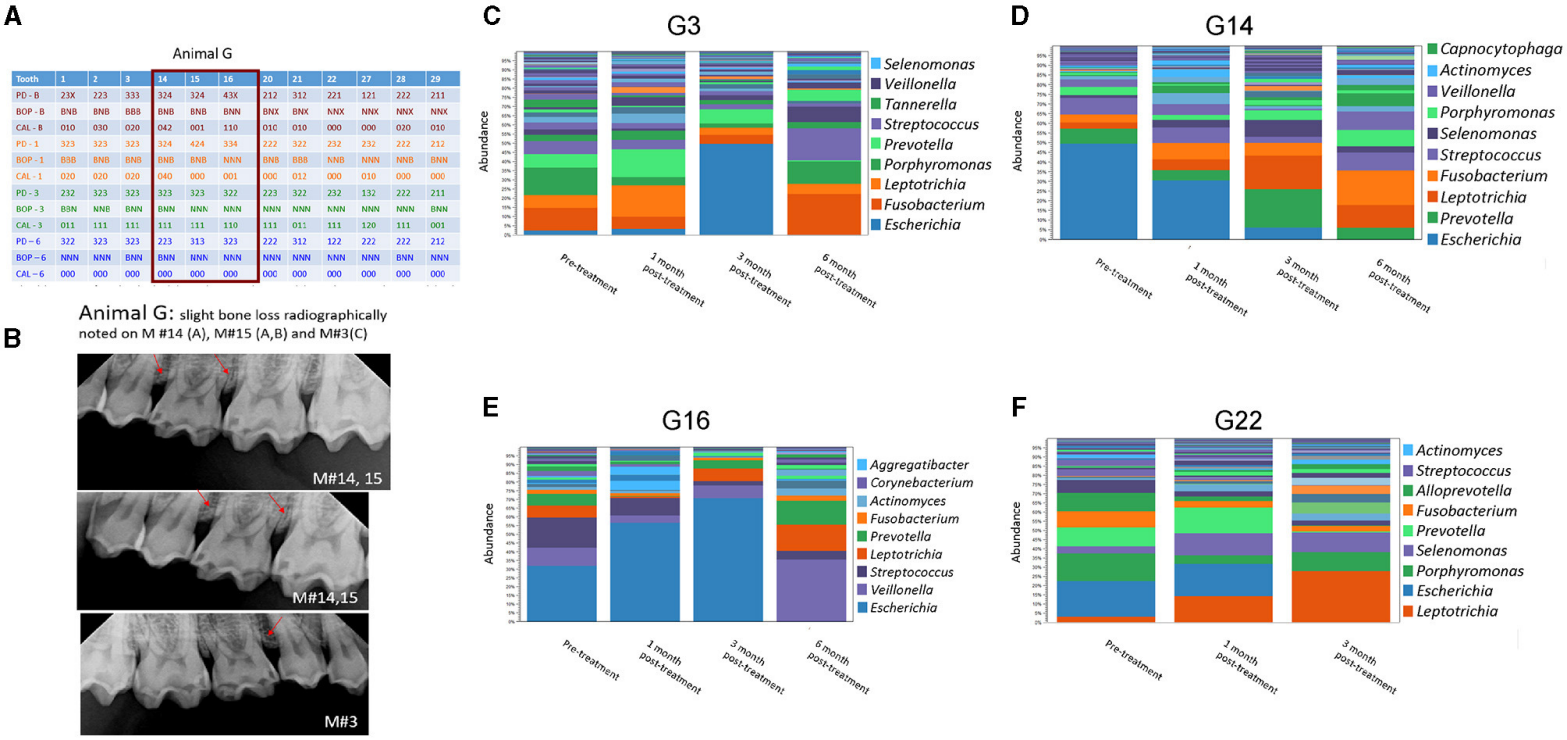
Effect of amixicile on subgingival microbiome of animal G. **(A)** Clinical characteristics of animal G at baseline (-B), 1-month post – amixicile (-1), 3-month post – amixicile (-3), 6-month post – amixicile (-6). PD – pocket depth (shown in mm), BOP– bleeding on probing (B – bleeding, N – no bleeding, X – not determined), CAL – clinical attachment loss. **(B)** Radiographs of selected sites taken at pre-treatment. **(C–F)** pre-treatment (before amixicile treatment, baseline), 1 – one month, 3 – three months, 6 – six months following amixicile treatment. Analysis at the genus level is shown for teeth 3 **(C)**, 14 **(D)**, 16 **(E)** and 22 **(F)** from Animal G.

**Figure 4:**
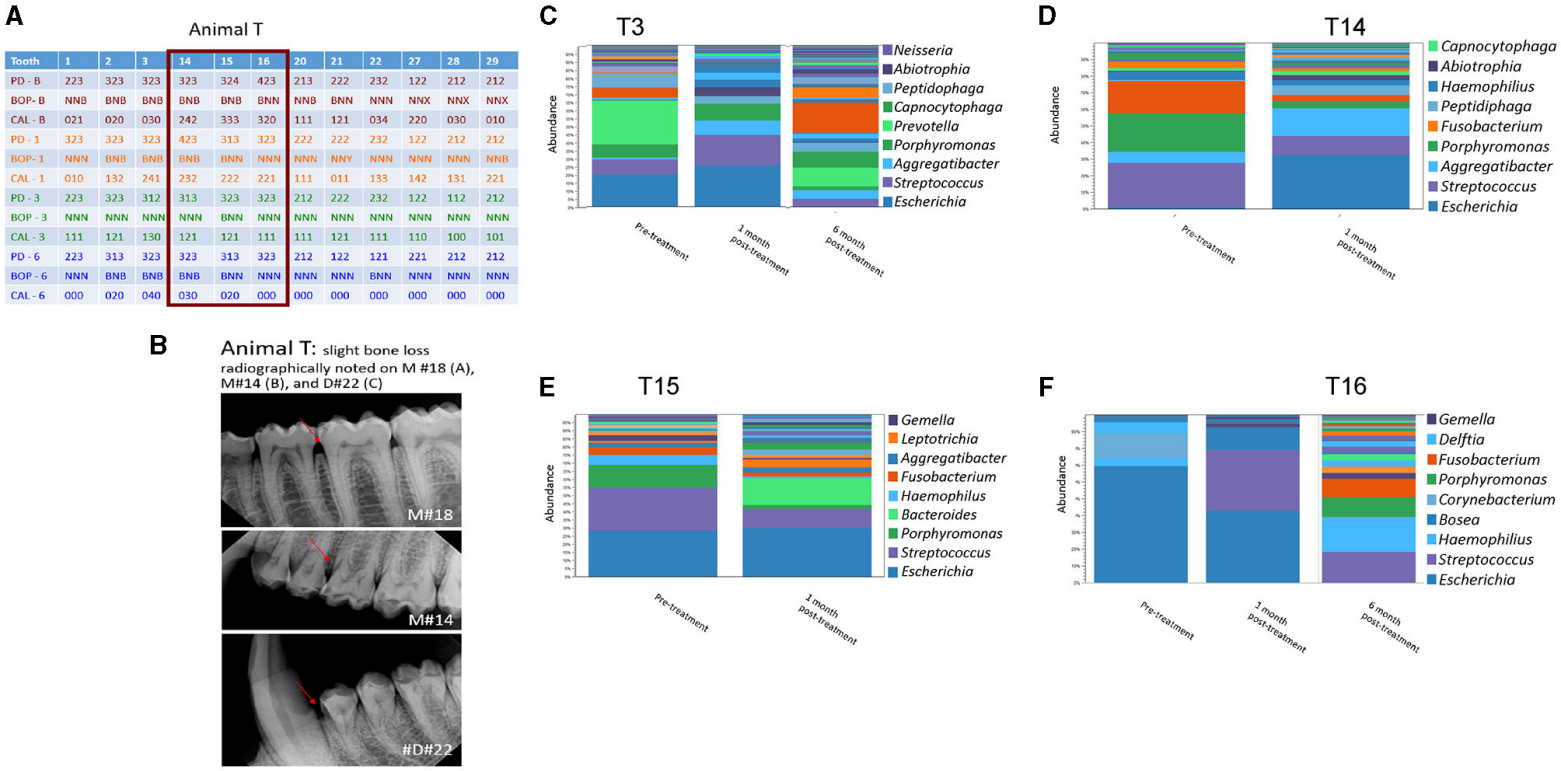
Effect of amixicile on subgingival microbiome of animal T. **(A)** Clinical characteristics of animal G at baseline (-B), 1-month post – amixicile (-1), 3-month post – amixicile (-3), 6-month post – amixicile (-6). PD – pocket depth (shown in mm), BOP– bleeding on probing (B – bleeding, N – no bleeding, X – not determined), CAL – clinical attachment loss. **(B)** Radiographs of selected sites taken at pre-treatment. **(C–F)** Pre-treatment (before amixicile treatment), 1 – one month, 3 – three months, 6 – six months (following amixicile treatment). Analysis at the genus level is shown for teeth 3 **(C)**, 14 **(D)**, 15 **(E)** and 16 **(F)** from Animal T.

The periodontal examination of both animals revealed mild periodontitis at several sites (Figures 1A,B, 3A,B, 4A,B). Animal G presented with localized areas exhibiting 2–4 mm CAL, but mostly 0–1 mm in the four exams (Figure 1A). The values for CAL in mm ranged: 0–4 (average 0.49) at baseline, 0–4 (average 0.34) posttreatment, 0–2 (average 0.78) at 3 months, and 0–3 (average 0.08) at 6 months. Animal T presented with a higher prevalence and generalized pattern of sites with CAL and thus displayed higher average values for CAL (Figure 1A). The values for CAL in mm ranged: 0–5 (average 1.30) at baseline, 0–4 (average 1.39) posttreatment, 0–4 (average 1.04) at 3 months, and 0–4 (average 0.40) at 6 months. In addition, both animals presented with PD of 1–4 mm at the baseline exam with localized interproximal PD of 4 mm and localized slight radiographic bone loss which corresponds to mild periodontitis for NHP [1]. We thus concluded that the clinical characteristics pointed to an acceptable level of localized mild periodontitis to be used in our study.

Average CAL for Animal G decreased from visit 1 to visit 2, and then increased by visit 3 and again decreased by visit 4. Animal T saw an initial increase in CAL at visit 2, followed by a decrease at visits 3 and 4. Both animals were below their initial average CAL by the 6-month examination (Animal G: 0.49 vs. 0.08; Animal T: 1.30 vs. 0.40; Figure 1A)

Gingival index was summarized by sextant (upper left, upper right, lower left, lower right). The average GI was calculated by averaging across the three raters and across all pictures. The trends for GI by animal and sextant are given in Figure 1C. Data for the GI of Animal G for the upper left sextant was not available. GI was relatively steady on the upper and lower left. The upper right initially decreased for Animal G and then increased steadily at 3- and 6-month examinations. Animal T increased slightly from initial to 3-month and then decreased by the 6-month exam. The lower right demonstrated a decrease from the initial to posttreatment and then increased at the 3-month exam. Between the 3-month and the 6-month examination, Animal T remained relatively steady and Animal G had a slight increase in GI scores.

Clinical findings across the visits are summarized in Figure 1B. Further statistical analyses were not performed due to the limited number of animals. Average PD for Animal G increased from visit 1 to visits 2 and 3 and then decreased by visit 4. Animal T saw an initial decrease at visit 2, followed by an increase at visit 3 and a decrease at visit 4. Both animals were at or below their initial average PD by the 6-month examination (Animal G: 2.00 vs. 1.99; Animal T: 1.93 vs. 1.73; Figure 1D). Both animals had an initial increase in the bleeding sites (Figure 1D) from visit 1 to visit 2, followed by a substantial improvement back to baseline at visit 3. By the 6-month follow-up, Animal G had an additional decrease in bleeding sites, but Animal T saw a slight increase. Although BOP decreased, the number of sites with plaque simultaneously increased progressively for both the animals from initial examination through the 6-month follow-up (Figure 1F). Both animals began with 0 plaque sites at baseline and finished with 96 for Animal G and 95 for Animal T.

For selected sites with microbiological analysis, 12 of the 36 sites (33.3%) in Animal G exhibited BOP at baseline. Similarly, in Animal T, there were four sites with BOP out of the 36 tested (11.1%). Following amixicile treatment, the number of sites with BOP remained similar to the baseline. Three months posttreatment there were no sites with PD of 4 mm in either animal. There were fourteen sites in Animal G and eleven sites in Animal T with a PD of 3 mm. Significantly, all the sites previously measuring 4 mm were reduced to 3 mm or less at 3 months posttreatment. At the final exam, no sites measured 4 mm, and only 11 sites measured 3 mm in each of the animals. BOP remained similar to baseline at the 3-month posttreatment exam. A significant reduction in BOP was noted in Animal G with four sites (11.1%), whereas Animal T experienced a rebound in BOP by the 6-month exam with eight sites (22.2%). The above observations suggest that a 2-week treatment with amixicile resulted in improvement in the BOP in Animal G from 33.3 to 11.1% (Figure 1E) and stability of PDs in Animal G and a reduction in PDs in Animal T from 1.93 mm at baseline to 1.73 mm at the 6-month exam (Figures 1B,D), all while both animals experienced a continuous dramatic increase of detectable plaque biofilm (Animal G: 51–96 sites; Animal T: 47–95 sites) (Figure 1F).

A more focused examination in Animal G shows that 4 mm PDs present at teeth #14, 15, and 16 at the baseline had reduced to 3 mm at 6 months posttreatment with amixicile (Figure 3A). Additionally, BOP for these teeth decreased from three sites to one at 3 months posttreatment and was maintained through the 6-month exam in Animal G. Similarly, in Animal T at teeth #14, 15, and 16, the number of sites with 4 mm PD was reduced from two to zero from baseline through 6-months posttreatment (Figure 4A), and BOP decreased to only one site at 3-months, but Animal T experienced a rebound between 3 and 6 months ending with three sites with BOP.

### Amixicile Reduces the Abundance of Anaerobic Bacteria in the Salivary Microbiome

Microbiological samples of saliva and subgingival plaque from 12 teeth on three facial sites (MB, B, DB) were collected and analyzed resulting in 36 sites per animal. Aliquots of pooled saliva collected during baseline and follow-up periodontal exams were used to isolate total DNA for 16S rRNA gene sequencing (Figure 2). Analysis of the data derived from Animal G at the genus level revealed that at baseline the dominant bacteria belonged to *Streptococcus* genus (Figure 2A). Abundant also were bacteria belonging to the *Haemophilus, Porphyromonas, Gemella*, and *Fusobacterium* genera. In Animal T bacteria belonging to the *Neisseria* genus were the most abundant ones (Figure 2C) along with bacteria belonging to the genera of *Porphyromonas, Streptococcus*, and *Gemella*. Having large proportions of anaerobic bacteria, and specifically, bacteria belonging to *Porphyromonas* and *Fusobacterium* justified the use of this model for testing of amixicile efficacy. Following treatment with amixicile, a reduction in anaerobic bacteria with a concomitant increase in aerotolerant ones was observed. Specifically, reduction in bacteria belonging to the genera *Porphyromonas, Fusobacterium*, and *Alloprevotella* was seen in Animal G. At the same time, an increase in *Haemophilus, Gemella*, and *Escherichia* was detected (Figure 2A). *Streptococcus* remained at the same level as in the pretreatment phase. In Animal T, we observed a reduction in the bacteria belonging to *Porphyromonas* (Figure 2C). However, a reduction in *Neisseria* was also observed. An increase in levels of aerotolerant bacteria belonging to the *Streptococcus, Haemophilus*, and *Gemella* genera were observed (Figure 2C). Overall, amixicile was effective in reducing the levels of anaerobic bacteria present in the salivary microbiome of an NHP model.

### Amixicile Reduces the Abundance of Anaerobic Bacteria in Gingival Pockets

Four sites from both animals were successfully surveyed for the composition of the oral microbiome at baseline and following amixicile treatment (Figures 3, 4). A Survey of the G3 site at the genus level in Animal G (Figure 3C) indicated that at the baseline *Porphyromonas* and *Fusobacterium* were highly dominant. Other abundant bacteria included *Leptotrichia, Prevotella*, and *Streptococcus*. Following amixicile treatment, a significant reduction in *Porphyromonas* and *Fusobacterium* levels was observed. This corresponded to an increase in levels of *Leptotrichia* and *Prevotella*. Analysis of the G14 site showed that at baseline, *Escherichia, Prevotella*, and *Streptococcus* were highly abundant genera (Figure 3D). Following amixicile treatment, levels of *Escherichia* and *Prevotella* were reduced whereas those of *Leptotrichia* and *Fusobacterium* increased. Baseline abundance of the G16 site displayed high levels of *Escherichia, Veillonella, Streptococcus, Leptotrichia, Prevotella*, and *Fusobacterium* (Figure 3E). Following treatment, the abundance of *Veillonella, Streptococcus, Leptotrichia, Prevotella*, and *Fusobacterium* were reduced whereas those of *Escherichia, Actinomyces*, and *Aggregatibacter* were elevated. The fourth site of Animal G, G22, displayed a high abundance of *Escherichia, Porphyromonas, Prevotella, Fusobacterium*, and *Alloprevotella* (Figure 3F). Following amixicile treatment, the levels of *Porphyromonas, Fusobacterium*, and *Alloprevotella* were reduced with an increase in *Leptotrichia, Selenomonas, Prevotella*, and *Actinomyces*. Interestingly, levels of *Streptococcus* were also reduced. Overall, in Animal G we observed a reduction in the levels of anaerobic bacteria and an increase in aerotolerant ones. There was significant variation in the microbial composition of the microbiome at the baseline and posttreatment.

Analysis of the subgingival microbiomes of Animal T, both at baseline and post-treatment showed a similar trend of reduced levels of anaerobic bacteria in favor of the aerobic ones. Specifically, the abundant genera of T3 were *Escherichia, Streptococcus, Porphyromonas*, and *Prevotella* (Figure 4C). Following amixicile treatment, the abundance of *Prevotella* was significantly reduced. Increased levels of *Escherichia, Streptococcus, Aggregatibacter*, and *Peptidiphaga* were observed (Figure 4C). At the second site T14, *Streptococcus, Porphyromonas*, and *Fusobacterium* were the dominant bacterial genera (Figure 4D). After amixicile treatment, the levels of *Porphyromonas, Fusobacterium*, and *Streptococcus* were significantly reduced, and this corresponded to an increase in the levels of *Escherichia, Aggregatibacter*, and *Peptidiphaga* (Figure 4D). The T15 site at baseline was abundant in *Escherichia, Streptococcus, Porphyromonas*, and *Haemophilus*. Following amixicile treatment, the levels of *Porphyromonas, Streptococcus, Haemophilus*, and *Fusobacterium* were significantly reduced with an increase in the levels of *Leptotrichia* and *Bacteroides* (Figure 4E). The final site analyzed for microbiome composition, T16, had high levels of *Escherichia* at baseline that got reduced with a concomitant increase in the levels of *Streptococcus* (Figure 4F). Similarly, as in Animal G, we observed a high variability between the microbial composition of the samples derived from different sites of Animal T. The common theme from the analyzed sites was the reduction in the abundance of anaerobic bacteria and increase in the levels of aerotolerant ones.

### The Effect of Amixicile on the Oral Microbiome Is Reversed After Several Months Post-treatment

We followed the microbiome changes of the animals up to 6-month posttreatment; two exams (3- and 6-months) were conducted where saliva and subgingival plaque from previously examined sites were collected and 16S rDNA was sequenced. The salivary microbiome of Animal G had increased in abundance in *Porphyromonas, Fusobacterium*, and *Leptotrichia*. Reduction in *Haemophilus, Streptococcus*, and *Gemella* was observed (Figure 2A). The composition resembled the baseline microbiome; however, higher levels of *Leptotrichia* were observed. In Animal T, the salivary microbiome had higher levels of *Haemophilus* and *Fusobacterium* after longer posttreatment intervals (Figure 2C). In the subgingival microbiome, an increase in the proportion of anaerobic bacteria was observed after longer posttreatment intervals (Figures 3, 4).

### Correlation Between the Microbiome and Clinical Characteristics

A significant clinical improvement as determined by CAL, PD, and BOP was observed at 3-month posttreatment and continued into the sixth month. These improvements were delayed with respect to the onset of reduction of anaerobic bacteria in the microbiome. At 6 months, despite some reversal of the abundance of anaerobic bacteria to the baseline levels, the clinical improvements still persisted despite the increased plaque burden.

## Discussion

The work presented here shows that the use of a 14-day treatment of amixicile at 40mg/kg/day is effective in reducing the clinical manifestations of periodontitis including reductions in CAL, PDs, and BOP. Overall, there is a notable decrease in the average values of CAL across visits for both animals. The reduction in values is noted at 3 months and continues at the 6-month exams. Along with a similar trend of decreasing PDs and BOP across visits in the presence of increasing plaque burden, the decreasing CAL may contribute to the microbiome shift following treatment with amixicile, which inhibits the growth of anaerobic bacteria.

A striking observation is that while all of these clinical indices were either decreasing or remaining stable, the plaque burden was steadily increasing. A possible explanation may be that although there is an increase in the number of bacteria, the balance could be shifted to a plaque rich in commensal and aerobic bacteria due to selective targeting of the anaerobic periodontal pathogens of amixicile. Reduction in more virulent periodontal pathogens may reduce the inflammatory response by the host, resulting in reduced levels of periodontal inflammation.

It is noteworthy to mention the conversion of the composition of the salivary and sulcular microbiomes from one prevalent in anaerobic bacteria to one with reduced levels of anaerobes and increased proportions of aerotolerant microorganisms. Saliva samples displayed a reduction in all anaerobes, such as *Porphyromonas, Fusobacterium*, and *Alloprevotella*, and with a concomitant increase in *Streptococcus, Haemophilus, Gemella*, and *Escherichia*, all of which are aerobes. Subgingival plaque samples showed similar alterations in microbial composition. Reduction of *Porphyromonas, Fusobacterium, Prevotella, Veillonella*, and *Alloprevotella*, all of which are anaerobes, was observed with a concomitant increase of known aerobes. These changes generally take place immediately posttreatment but return to baseline levels by 6 months.

There was a great degree of variability in microbial composition between individual sites, which would be expected but makes comparison difficult. Microbial composition likely varies based on the depth of sulcus as well as environmental factors such as chewing function and self-cleansability and exhibits site variability. The lack of diversity in the microflora in Animal T, site #16 at baseline is noteworthy (Figure 4F). This site had very little biofilm and was periodontally healthy at the initial exam, and thus the DNA had to be amplified many times to yield data resulting in a favored representation of highly abundant bacteria and uncertain representation of bacteria in low abundance. As the biofilm continued to accumulate, a more diverse flora is noted at the 6-month exam.

It is noteworthy that we used animals with naturally occurring periodontitis rather than ligature-induced one [13]. Comprehensive clinical assessment of the periodontal status and indices were supported by the radiographs to diagnose periodontitis and thus extended the assessments beyond what was used in previous studies [12, 13]. Limitations include a small sample size as the expense of acquiring and maintaining these animals is significant. Given that only two subjects were available, both animals received treatment resulting in no control group in the study. The animals were 15 and 16 years old, respectively, and thus qualified as adults [12, 13]. The animals also exhibited only mild periodontitis in a small number of sites. Another limitation is that the concentrations of amixicile or any inflammatory markers in the GCF or serum were not measured. While the microbiological data would support action of amixicile, it can not be determined how well it was able to localize to the gingival sulcus. In our study, V3-V4 primers were used which allow for broader phylogenetic coverage than V1–V2 primers. This is both an advantage and a disadvantage as these primers target 16S rDNA, but different strains of bacteria cannot be distinguished using this method [14].

In conclusion, amixicile selectively inhibits the growth of known anaerobic periodontal pathogens including *Porphyromonas spp., Fusobacterium spp.*, and *Prevotella spp*. causing a reduction in clinical indices of periodontal inflammation for a period of up to 6 months. If amixicile continues to demonstrate success at selectively targeting periodontal pathogens with little side effects, it may offer an alternative to current antimicrobial options, such as the replacement of the amoxicillin/metronidazole combination. In our study, a 14-day course resulted in a significant reduction in pathogenic bacteria with a rebound of the microbes occurring between 3 and 6 months. With no known side effects thus far, amixicile may be considered as a suitable candidate for antimicrobial treatment of periodontitis. Further research at a larger scale is needed to bring the drug closer to the possibility of a clinical trial in humans.

## Data Availability Statement

The datasets presented in this study can be found in online repositories. The names of the repository/repositories and accession number(s) can be found below: NCBI SRA; PRJNA753125.

## Ethics Statement

The animal study was reviewed and approved by Virginia Commonwealth University (VCU) Institutional Animal Care and Use Committees (IACUC) AD10001255.

## Author Contributions

QG and DL performed most of the experiments, analyzed the data, prepared figures for the manuscript, and contributed to the writing of the manuscript. BB prepared the libraries for sequencing of the microbiome. JD was instrumental in planning the clinical parts of the study design, clinical examinations of the animals, assisted with analysis and interpretation of data, and writing of the manuscript. JL conceived the idea for this project, assisted with clinical examination and performed bioinformatics analysis of the microbiome, and supervised all of the work reported in this manuscript. JL wrote the first draft as well as revised the manuscript. PH provided the amixicile as well as helpful comments for this work. All the authors reviewed the manuscript. All authors contributed to the article and approved the submitted version.

## Funding

A portion of the work was supported by the NIH NIDCR K18DE031096 and the VCU's CCTR Endowment Fund to JL, the VCU's School of Dentistry Alexander Fellowship to DL, as well as funds provided by the Philips Institute.

## Conflict of Interest

The authors declare that the research was conducted in the absence of any commercial or financial relationships that could be construed as a potential conflict of interest.

## Publisher's Note

All claims expressed in this article are solely those of the authors and do not necessarily represent those of their affiliated organizations, or those of the publisher, the editors and the reviewers. Any product that may be evaluated in this article, or claim that may be made by its manufacturer, is not guaranteed or endorsed by the publisher.

## Supplementary Material

Supplemental Figure 1 Experimental design showing the timeframe of sample collection and clinical exams followed by the flowchart of sample processing and analysis.

Supplemental Figure 2 Clinical photos to support the presence of periodontal disease.
