# Supplementary material for: Non-human Primate Macaca mulatta as an Animal Model for Testing Efficacy of Amixicile as a Targeted Anti-periodontitis Therapy
Source: Front Oral Health. 2021 Nov 5;2:752929. doi: 10.3389/froh.2021.752929 (PMC8757802; doi:10.3389/froh.2021.752929)
Supplement: Supplemental Figure 1 — Experimental design showing the timeframe of sample collection and clinical exams followed by the flowchart of sample processing and analysis. [file Data_Sheet_1.pdf]

Supplementary Material:

Supplemental Figure 1. Experimental design showing the timeframe of sample collection and clinical the exams followed by flowchart of sample processing and analysis.

Supplemental Figure 2. Clinical photos to support presence of periodontal disease.

Supplementary Fig. 1. Experimental design.

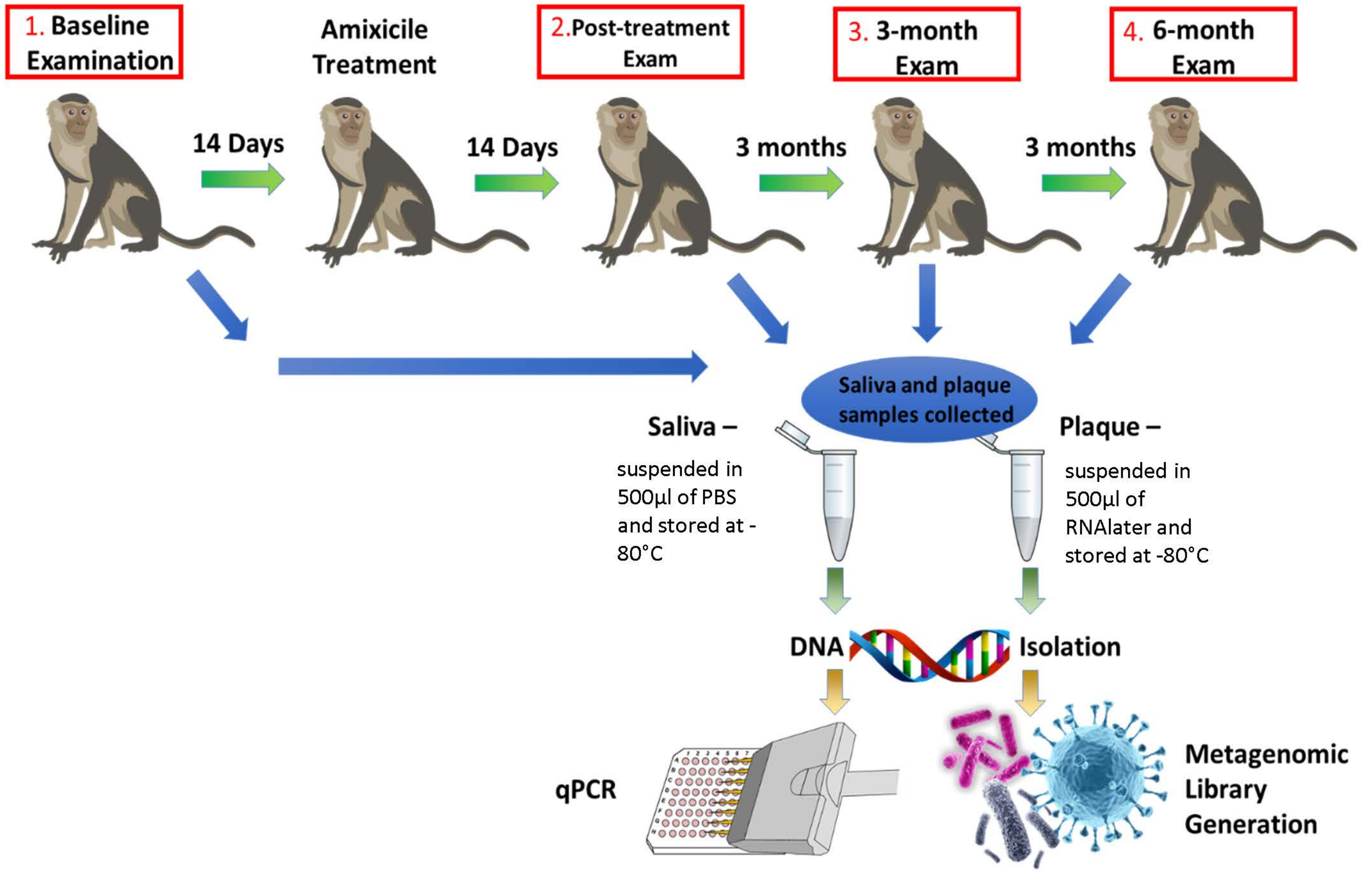

Created by Universal Document Converter

Supplementary Fig. 2. Clinical photos taken to assess the Gingival Index values

Animal T: Upper Left including #14,15 at visit 4

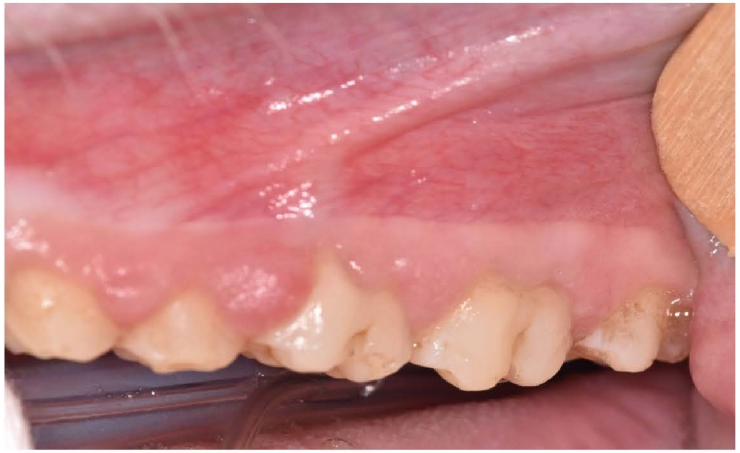

Animal G: Upper Right including #3 at visit 4

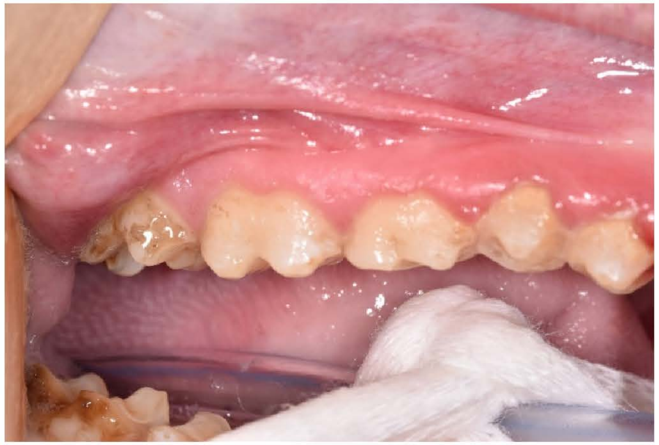

Animal T: Upper Right including #3 at visit 3

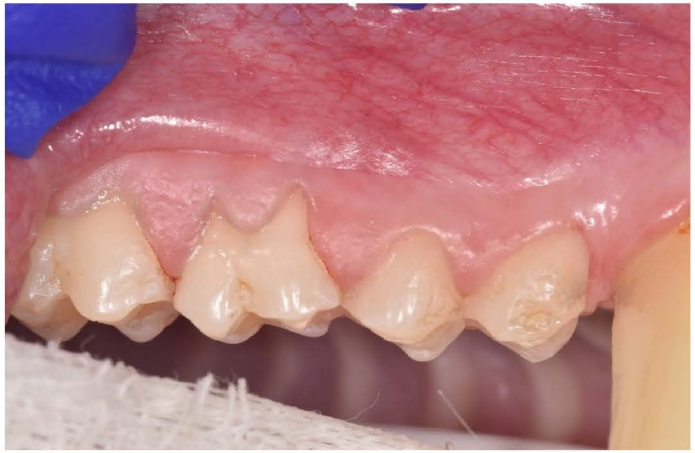

Animal G: Upper Left including #14 at visit 2

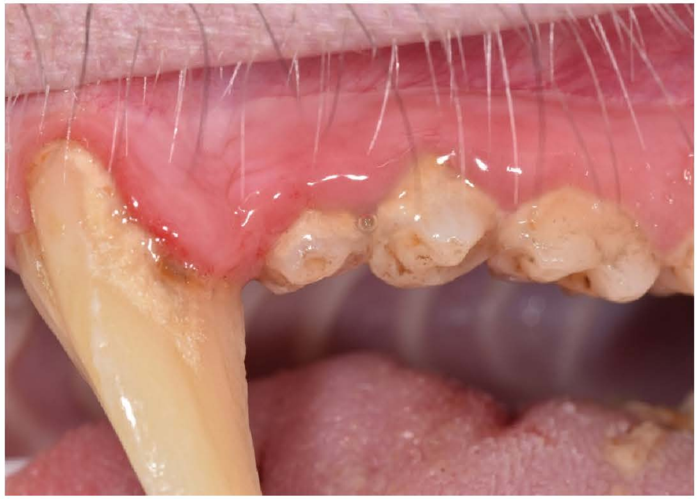

Created by Universal Document Converter
